# Prognostic significance of baseline thyroid variables in IDH-wildtype glioblastoma patients treated with regorafenib

**DOI:** 10.1007/s11060-023-04408-1

**Published:** 2023-08-10

**Authors:** Aaron Lawson McLean

**Affiliations:** Department of Neurosurgery, Jena University Hospital – Friedrich Schiller University Jena, Am Klinikum 1, 07747 Jena, Germany

**Keywords:** Thyroid function, Regorafenib, Glioblastoma, IDH Mutation, Treatment response, Multicenter Study

To the Editor,

I read with interest the article by Caccese et al., “Association between thyroid function and regorafenib efficacy in patients with relapsed wild-type IDH glioblastoma: a large multicenter study” [1]. The attempt to decipher the predictive markers for regorafenib response in glioblastoma patients is an insightful approach towards individualized treatment regimens. However, I would like to address certain areas where additional discussion and future research could further strengthen the implications of this study.

Firstly, the statistical methodology used to identify the non-linear relationship between the fT3/fT4 ratio and survival outcomes merits further explanation. A comprehensive detailing of the Cox regression models, the incorporation of interaction terms, and the transformation of thyroid function variables would provide readers with a more nuanced understanding of the methodology.

While the study duly noted baseline corticosteroid levels, the retrospective nature inherently poses limitations. The exclusion criteria of patients without baseline thyroid function values could potentially introduce selection bias. Patients with missing baseline thyroid function values might represent a distinct cohort with inherent differences that could skew the results.

The study’s intriguing finding regarding the correlation between lower baseline TSH values and higher rates of disease progression to regorafenib invites further investigation into the physiological basis of this relationship. The underlying mechanisms driving this correlation might be rooted in the influence of TSH on cell proliferation, apoptosis, and other processes vital in tumor biology [2].

While it is compelling to consider baseline TSH as a predictor of regorafenib activity, it is crucial to remember that correlation does not equate to causation. It is plausible that TSH levels might be an indirect marker of overall health status, with altered levels observed in more unwell patients who are less likely to respond to therapy [3].

Additionally, potential confounders that could impact thyroid function such as iodine status, autoimmunity, and non-thyroidal illness syndrome have not been addressed. The effects of these variables on the study’s outcomes add another layer of complexity to the interpretation of the results.

In conclusion, the study by Caccese et al. is a significant contribution to our understanding of the complex interplay between thyroid function and treatment outcomes in glioblastoma. While the findings are promising, they underline the need for further prospective studies that incorporate potential confounders and validate the predictive value of thyroid function parameters in the treatment response.

Sincerely,

Aaron Lawson McLean.
